# A distributed query execution engine of big attributed graphs

**DOI:** 10.1186/s40064-016-2251-0

**Published:** 2016-05-23

**Authors:** Omar Batarfi, Radwa Elshawi, Ayman Fayoumi, Ahmed Barnawi, Sherif Sakr

**Affiliations:** King Abdulaziz University, Jeddah, Saudi Arabia; Princess Nourah Bint Abdulrahman University, Riyadh, Saudi Arabia; University of New South Wales, Sydney, Australia; King Saud bin Abdulaziz University for Health Sciences, Riyadh, Saudi Arabia

## Abstract

A graph is a popular data model that has become pervasively used for modeling structural relationships between objects. In practice, in many real-world graphs, the graph vertices and edges need to be associated with descriptive attributes. Such type of graphs are referred to as *attributed graphs*. G-SPARQL has been proposed as an expressive language, with a *centralized* execution engine, for querying attributed graphs. G-SPARQL supports various types of graph querying operations including reachability, pattern matching and shortest path where any G-SPARQL query may include value-based predicates on the descriptive information (attributes) of the graph edges/vertices in addition to the structural predicates. In general, a main limitation of centralized systems is that their vertical scalability is always restricted by the physical limits of computer systems. This article describes the design, implementation in addition to the performance evaluation of *DG-SPARQL*, a *distributed*, hybrid and adaptive parallel execution engine of G-SPARQL queries. In this engine, the topology of the graph is distributed over the main memory of the underlying nodes while the graph data are maintained in a relational store which is replicated on the disk of each of the underlying nodes. DG-SPARQL evaluates parts of the query plan via SQL queries which are pushed to the underlying relational stores while other parts of the query plan, as necessary, are evaluated via indexless memory-based graph traversal algorithms. Our experimental evaluation shows the efficiency and the scalability of DG-SPARQL on querying massive attributed graph datasets in addition to its ability to outperform the performance of Apache Giraph, a popular distributed graph processing system, by orders of magnitudes.

## Introduction

In this era, we are witness continuous expansion and integration of computation, networking, digital devices and data storage systems in a way that provided a rich platform for the explosion in big data as well as the means by which big data are produced, stored, processed and analyzed. In practice, there exist various modern big data applications where data are intuitively and naturally modeled as big graphs including social networks, spatial road networks, protein interaction networks, neural networks and the Internet of Things (Faloutsos et al. 1999; Kleinberg et al. 1999). For example, Facebook reported that, during the first quarter of 2015, it had an average of 1.44 billions monthly active users.^1^ Therefore, it has become very crucial for several applications to have the ability of efficiently store, query and analyze these big graphs (Sakr and Pardede 2011).

Attributed graph (Ehrig et al. 2004) is a variant graph data model where each node^2^ is identified with a unique identifier and labeled with a string. Each edge in the attributed graph is also identified with a unique identifier and labeled with a string. In addition, each edge connects a source node to a destination node. In attributed graphs, each node or an edge can be associated with a collection of key/value pairs that represent its descriptive information or properties. Given a large attributed graph that includes billions of edges and nodes (e.g., bibliographic network, social network) with their descriptive information, one of the fundamental challenges is on how to efficiently query and analyze these big graphs.

In practice, querying datasets which are represented using any kind of data models (e.g., Relational, XML, Graph) typically involves two main steps: query representation and formulation using a query language (e.g., SQL for relational model, XPath for XML) and efficient evaluation of the formulated queries using a querying execution engine. Although SQL is a popular and standard query language for the relational model, it is not adequate for graph querying purposes as it requires users to reason in terms of tables and join operations between them instead of the intuitive reasoning of graph as a group of vertices and edges that link them. Therefore, in the general context of the graph data model, a number of graph querying languages has been proposed such as: PQL (Leser 2005), GraphQL (He and Singh 2008), SPARQL (Prud’hommeaux and Seaborne 2008) and Cypher (2015). G-SPARQL (Sakr et al. 2012) has been proposed as an expressive language with design focus on querying attributed graphs. The language supports the formulation of various kinds of graph querying operations including reachability, shortest path and pattern matching queries. In G-SPARQL, each query may include value-based predicates on the attributes of the graph edges/vertices in addition to the structural predicates. Sakr et al. (2014) presented a *centralized* execution engine for G-SPARQL queries which identifies parts of the query plan (sub-plans) to be evaluated using the underlying relational store via SQL queries while the evaluation of other sub-plans of the main query plan are executed via indexless main memory-based graph traversal algorithms. In general, one of the fundamental limitations of centralized systems is that their performance is bounded by the computing resources which can be allocated to a single machine. In addition, centralized systems can only be scaled vertically by adding more computing resources to the underlying machines. However, the vertical scalability of centralized systems is always restricted by the physical limits of computer systems. On the other hand, a distributed system represents a set of autonomous nodes, each with their computing resources (e.g., memory, disk), that cooperate to perform computations and exchange data as messages via a network. In practice, one of the main advantages of distributed data processing systems is that they can scale to nearly arbitrarily increasing data sizes by effectively leveraging horizontal scalability where additional computer resources (cooperating nodes) can be added easily.

With the increasing size of big graph datasets and the growing needs and popularity of interactive querying systems over these graphs, it becomes crucial to manage large graphs in distributed environments that can support query execution with low latency. In this article, we present *DG-SPARQL*, short for *D*istributed *G-SPARQL*, a distributed query execution engine which takes the evaluation of G-SPARQL queries to the next level in terms of performance and scalability. In particular, DG-SPARQL is designed for handling large and distributed attributed graphs and overcoming many of the challenges and limitations of centralized query engines. In DG-SPARQL, the topology of the graph is distributed over the main memory of the underlying nodes while the graph data are maintained in a relational store which is replicated on the disk of each of the underlying nodes (Hammoud et al. 2015). Similar to the centralized implementation of G-SPARQL, DG-SPARQL evaluates parts of the query plan via SQL queries which are pushed to underlying RDBMS nodes while other parts of the query plan are evaluated via indexless main memory-based graph traversal algorithms, as needed. However, DG-SPARQL applies selectivity-based query processing that exploits the estimation of predicate selectivities to parallelize and optimize the query evaluation process using the divide-and-conquer strategy for generating the query plans. In particular, the number of used RDBMS nodes for each query varies and is determined based on a defined cost model. Thus, DG-SPARQL combines the advantages of the efficient data storage and query execution features of relational stores, the efficiency of main memory graph traversal operations in addition to the efficiency and scalability of distributed systems. The main contributions of this article can be summarized as follows:

- We present the design and implementation of *DG-SPARQL*, a full-fledged distributed and parallel G-SPARQL query execution engine of big attributed graphs. In DG-SPARQL, the graph topology is loaded into the distributed main memory of the computing cluster while the graph data is replicated on a relational store at each node. In practice, providing scalable execution engines of expressive query languages for big attributed graphs expands the effectiveness of analyzing and understanding real world graphs and enriches the variance on the kinds of questions which could be answered via graph querying systems.
- DG-SPARQL adopts a rule-based query optimizer to split the query plan among the main memory and relational components of the execution engines. In addition, it adaptively selects different numbers of the underlying relational nodes, for each query, for executing the SQL-based parts of the execution plan using selectivity estimation techniques and a cost model.
- We demonstrate the efficiency and scalability of DG-SPARQL via an extensive set of experiments that use big synthetic and real graph datasets in addition to a comparison with Apache Giraph, a popular distributed graph processing system.

The remainder of this article is organized as follows. Background information about attributed graphs and G-SPARQL query language are provided in “Background” section. Details of the distributed hybrid representation of DG-SPARQL for the attributed graphs are presented in “Distributed hybrid representation of the attributed graphs” section while the details of the distributed query execution engine are presented in  “Distributed query execution engine” section. The results of our performance evaluation are presented in “Experimental evaluation” section. The related work on graph querying systems is reviewed in “Related work” section before we finally conclude the article in “Conclusion” section.

## Background

Herewith, we introduce the main concepts that form the groundwork for our presented system: attributed graph and the G-SPARQL query language.

### Attributed graphs

In many real applications, it is of high practical value that the graph edges and nodes get associated with descriptive information (attributes) in the form of key-value pairs. This type of graphs are referred to as *attributed graphs*. Formally, an *attributed graph* is denoted as ($N, E, L_n, L_e, F_n, F_e, \Gamma _n, \Gamma _e$) where:

- *N* defines the set of graph nodes that represent the application objects.
- $E \subseteq N \times N$ defines the set of edges joining two graph nodes and represent the structural relationships between the application objects.
- $L_n$ is the set of labels for the graph nodes.
- $L_e$ is the set of labels for the graph edges.
- $F_n$ is a function $N \rightarrow L_n$ that associates labels with the graph nodes.
- $F_e$ is a function $E \rightarrow L_e$ that associates labels with the graph edges.
- $\Gamma _n =\{a_1, a_2,\ldots , a_x\}$ is a set of *x* attributes that can be associated with any graph node $(n) \in N$. In particular, each node $n \in N$ can be associated with a vector of key/value pairs $[a_1(v_1),\ldots , a_m(v_m)]$ where $a_j(v_j)$ represents the attribute value of node *n* on attribute $a_j$.
- $\Gamma _e =\{b_1, b_2,\ldots , b_y\}$ is a set of *y* attributes that can be associated with any edge $(e) \in E$. In particular, each edge $e \in E$ can be associated with a vector of key/value pairs $[b_1(e_1),\ldots , b_n(e_u)]$ where $b_k(e_k)$ represents the attribute value of edge *e* on attribute $b_k$.

**Fig. 1:**
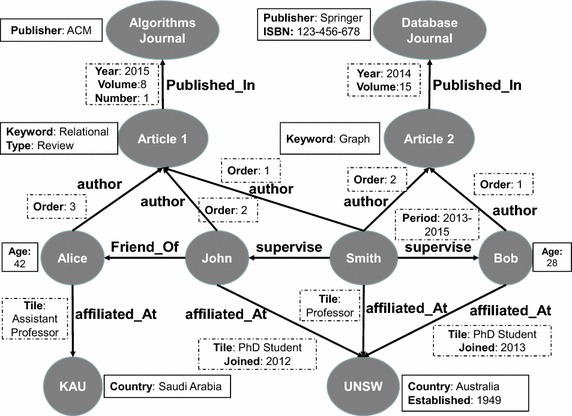
A sample attributed graph for bibliographic network

Figure 1 illustrates an example of a snippet of an attributed graph of a bibliographic network where each node represents an object instance (e.g. *article*, *author*, *scientific journal*, *affiliation*) and each edge represents a structural relationship (e.g. *supervise*, *friend_Of*, *author*, *affiliated_At*, *published_In*) between two graph nodes. Additionally, the various types of graph nodes are described with different attributes (e.g. *type*, *keyword*, *publisher*, *volume*, *country*), denoted with solid rectangles, while another set of attributes (e.g. *title*, *order*, *year*), denoted with dashed rectangles, are used to describe their associated graph edges.

In the general context of large graphs, there are popular kinds of graph querying operations including *reachability queries* that check the existence of a path between two nodes in the large graph, *shortest path queries* that returns the path, if it exists, with the smallest number of hops between any two nodes in the large graph and *pattern matching queries* that look for the occurrence(s) of a pattern-based subgraph in the large graph. In practice, in the context of large attributed graphs, it is common for many users to have the need to formulate queries that involve more than one of these graph querying operations. In addition, they commonly need to express filtering conditions (predicates) on the associated descriptive information (attributes) of the graph edges/nodes. Using the sample attributed graph of the bibliographic network illustrated in Fig. 1, samples of such queries are:

(SQ1): *Structural pattern matching query with filtering conditions on the values of the attributes of the graph edges and nodes*.
  Search for the names of two authors, A and B, where AsupervisesB, both of A and B are affiliated_At UNSW, the age of B is greater than 25, the title of A at UNSW is ’Professor’ and B joined UNSW after 2010.
(SQ2): *Structural reachability query with filtering conditions on the values of the attributes of the graph edges and nodes*.
  Search for the names of two authors, A and B, who are connected with a path which is less than or equal 4 steps (edges) where the age of A is greater than 25 and the age of B is greater than 35.
(SQ3): *Structural reachability query combined with structural pattern matching in addition to filtering conditions on the values of the attributes of the graph edges and nodes*.
  Search for the names of two authors, A and B, who are connected via a sequence of edges (path) which is less than or equal 3 steps (edges) where the age of A is greater than 25, the age of B is greater than 35, A is affiliated_At KAU with title of Assistant Professor and Y joined UNSW after 2010.
(SQ4): *Structural pattern matching query combined with structural reachability query with filtering conditions on the attributes of the edges of the retrieved path by the reachability query*.
  Search for the names of two authors, A and B, who are connected via a sequence of edges (path) which is less than or equal 3 steps (edges) where the age of A is greater than 25, the age of B is greater than 35 and no one of the authors in the connecting path between A and B has the tile of PhD student.

### G-SPARQL query language

The first step on querying any kind of data is to formulate the user queries using an adequate expressive query language. The SPARQL query language has been recognized as the official W3C language for querying RDF graphs (Prud’hommeaux and Seaborne 2008). In general, there are some fundamental differences between the attributed graph model and the RDF data model. For instance, the RDF data model uses graph edges to model both of the attribute/value pairs of the graph vertices, similar to the way of modeling the structural relationship with the other graph vertices. In addition, while the attributed graph considers edges as a first class citizen that can be directly associated with descriptive attributes, the RDF data model does not directly support associating the graph edges by descriptive attributes. However, a *reification* mechanism can be used to indirectly achieve this goal by relying on a nesting mechanism, auxiliary nodes. In practice, this mechanism is commonly referred to as “*The RDF Big Ugly*” (Powers 2003) as it can dramatically increase the graph size and consequently affects the query processing time. In addition, this solution is much less intuitive when it comes to the user on expressing his queries. Furthermore, some graph query operations which are of popular interest in the domain of large attributed graphs (e.g., *shortest path*) are not often considered as the main attention within the context of the RDF data model.

**Fig. 2:**
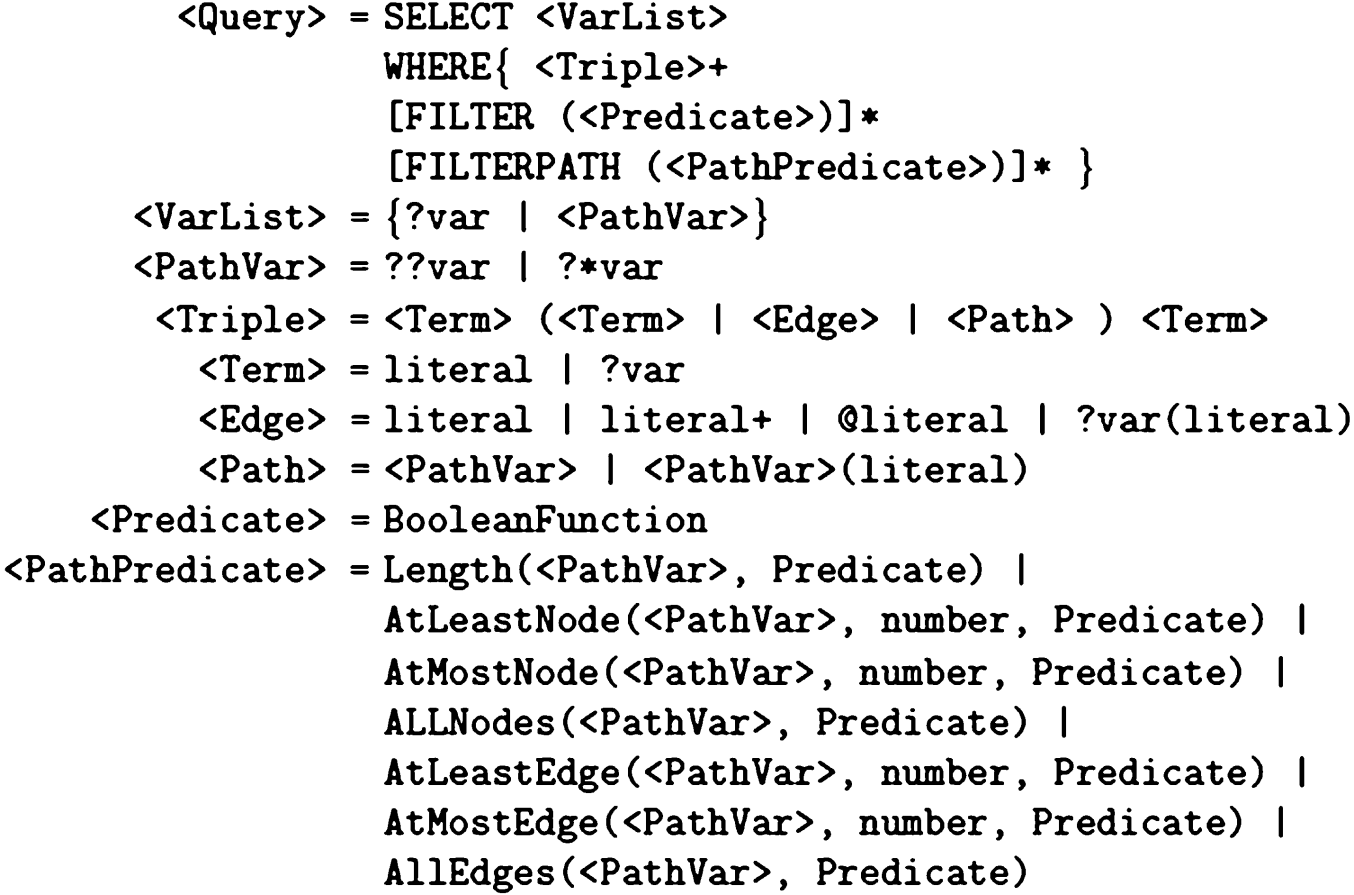
The grammar of *G-SPARQL* language (Sakr et al. 2012)

*G-SPARQL* (Sakr et al. 2012) has been introduced as a concise extension of the SPARQL query language which is mainly focusing on addressing the user requirements for querying large attributed graphs. In principle, one of the main design decisions of the G-SPARQL query language is to keep the required extensions, on SPARQL, for the purpose of querying property graphs minimum. As a result, the space and complexity of issuing a query using DG-SPARQL is very similar to the SPARQL language. An additional advantage of this design decision is that the learning curve for G-SPARQL should be minimum for any user who is familiar with the SPARQL language. In particular, G-SPARQL relies the fundamental graph matching facility of the SPARQL language. however, it introduces language constructs for defining predicates on the values of the attributes of the graph edges or nodes that are specified in the query pattern. G-SPARQL also provide language constructs that support various and rich forms of querying graph paths (sequence of edges) that facilitates the formulation of shortest path queries and reachability queries between the graph nodes (e.g., graph edge traversals with filters on the values of the edge attributes). Furthermore, G-SPARQL enables expressing filtering predicates on the queried path patterns.

The grammar of the *G-SPARQL* language is illustrated in Fig. 2. For the full details on the syntax and semantics of *G-SPARQL*, the readers are referred to Sakr et al. (2012). In the following we show the G-SPARQL code examples that formulate the sample queries on the attributed graph of “Attributed graphs” section. We start by illustrating the following G-SPARQL query syntax which formulates the semantics of the example query (SQ1) in “Attributed graphs” section.

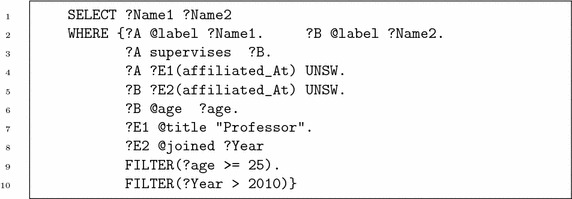



In this example, Line 3 formulates the structural supervise relationship between the the two target authors. The query triples in Lines 4 and 5 ensure that both authors are connected with a graph edge that represents the affiliated_At relationship with UNSW. Lines 6 and 9 represent a filtering condition on the age attribute of the graph node which represents the author B. Line 7 represents a boolean predicate on the title attribute of the graph edge which represents the affiliated_At relationship of author A. Lines 8 and 10 represent a filtering condition on the Year attribute of the graph edge which represents the affiliated_At relationship of author B.

The formulation of the semantics of the example query (SQ2) in “Attributed graphs” section is represented with the following G-SPARQL query syntax.

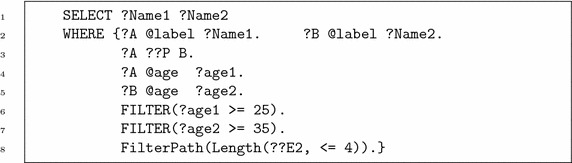



In this example, Line 3 formulates a structural reachability query between the the target author nodes. Line 8 represents a filter condition on the reachability query to filter out any reachable paths with more than 4 steps (edges). Lines 4 and 6 represent a filtering condition on the age attribute of the graph node which represents the author A while lines 5 and 7 represent a filtering condition on the age attribute of the author B.

Finally, the following G-SPARQL query syntax formulates the semantics of the example query (SQ3).

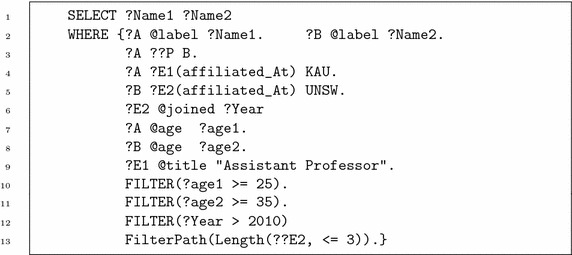



Line 3 formulates a structural reachability query between the the target author nodes with a filter condition on the reachability query to filter out any reachable paths with more than 3 steps (edges), Line 13. Line 4 represents a structural predicate the ensures that author A is connected with affiliated_At relationship to KAU, with boolean predicate on the title attribute of the graph edge, Line 9. Line 5 represents a structural predicate that ensures that author B is connected with the affiliated_At relationship to UNSW while Lines 6 and 12 formulate the filtering condition on the joined attribute of the graph edge which represents the affiliated_At relationship. Lines 7 and 10 formulate a filtering condition on the age attribute of the graph node which represents the author A while lines 8 and 11 represent a filtering condition on the age attribute of the author B.

## Distributed hybrid representation of the attributed graphs

In general, over several decades, relational model and relational database management systems (RDBMSs) have been recognized as the most widely used technology for data-intensive storage and querying applications. RDBMSs are well-known for their ability to support very efficient query engines that make use of various efficient data indexing mechanisms in addition to advanced query optimization techniques (e.g. join ordering, cost-based query processing). Therefore, several techniques and systems have been utilizing the efficiency of the relational model and RDBMSs for storing and querying various more sophisticated data models including XML (Gou and Chirkova 2007; Grust et al. 2004), RDF (Sakr and Al-Naymat 2009) and graphs (Sakr 2009; Sakr and Al-Naymat 2010). On the other hand, relational databases have shown to be inefficient for querying operations that involves recursive access or looping for significant numbers of rows via performing various expensive join queries that may lead to considerably huge intermediate results. Therefore, in the context of the graph model, performing traversal operations over the vertices and edges of graph-structured data which are stored in relational database turns to be time-inefficient because of the extensive number of required join operations plus the very expensive I/O disk access cost. Hence, it becomes more efficient to utilize main memory-based techniques to perform graph querying operations that involves heavy traversals on the graph topology (i.e, nodes and edges).

In our approach, we follow the hybrid Disk/Memory mechanism for managing attributed graphs which is presented by Sakr et al. (2012). In this mechanism, the data of the graph are maintained in a relational store while the topology of the graph is loaded into the main memory via a native pointer-based data structure for the sake of performing efficient graph traversal operations. In particular, a fully decomposed storage model (DSM) (Abadi et al. 2007; Copeland et al. 1985) is employed to store the attributed graph where each node and edge in the graph is assigned a unique identifier then the attributed graph is modeled using $M + N$ 2-column tables and *P* 3-columns tables where *M* represents the number of unique attributes of the graph nodes, *N* represents the number of unique attributes of the graph edges and *P* is the number of unique relationships that occur among the graph nodes. Each of the ($M + N$) 2-columns tables collects the values for one attribute where it stores the node identifier (in the *M* tables) or the edge identifier (in the *N* tables) on the first column while the second column (*Value*) maintains the value of the associated attribute. The *P* 3-columns tables maintain the information of the graph topology where each table collects the information of all graph edges that models a specific relationship. Specifically, in these tables, each row describes the information of a graph edge via 3 attributes: the edge identifier (*eID*), the identifier of the source node of the edge (*sID*) and the identifier of the destination node (*dID*).

**Fig. 3:**
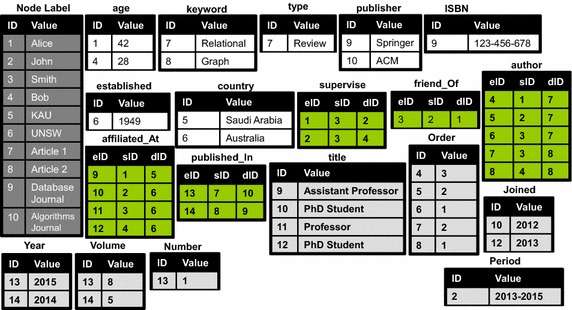
DSM relational encoding of attributed graph of Fig. 1

Figure 3 illustrates the relational representation for the sample attributed graph of Fig. 1 using the described fully decomposed storage model. In this figure, the table Node Label encodes *all* the graph nodes using their identifiers and labels. The 2-column tables with the white background $\{$age, keyword, type, publisher, ISBN, established, country$\}$ encodes the key/value pairs of the attribute information of the graph nodes. The 3-column tables $\{$supervise, friend_Of, author, affiliated_at, published_In$\}$ encodes the graph edges with the structural information of connecting the graph nodes. The 2-column tables with the dark background $\{$title, order, joined, year, volume, number, period$\}$ encodes the attribute information of the graph edges. Each of these encoding tables is indexed on its ID column with the aim of enabling efficient merge join operations for retrieving attributes of the same node/edge. Additionally, for each encoding table, a partitioned B-tree index (Value, ID) is used with the aim of enabling efficient execution of the value-based predicates on the attributes of the graph vertices or edges (Graefe 2003). For the graph topology, a native pointer-based main memory encoding is used to represent the information of the *P* tables which maintain the structural information of the graph edges. In practice, the *P* tables encode the mandatory information for performing index-free traversal operations on the graph topology [e.g., depth-first search (DFS) (Korf 1985) or breadth-first search (BFS) (Zhou and Hansen 2006)].

**Fig. 4:**
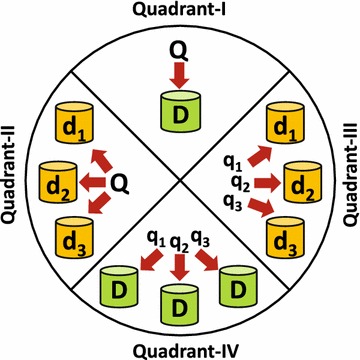
The four paradigms for building data storage and querying systems Hammoud et al. (2015)

In general, there are two main options for realizing scalability for data storage and querying application in order to cope with increasing data size and applications workloads: (1) *Vertical Scalability*: This option is implemented via allocating a bigger machine with more computing resources (e.g., CPU, Disk, Main Memory). (2) *Horizontal Scalability*: This option is implemented by distributing/replicating the data across multiple machines. In practice, the option of vertical scalability has the main limitation that its scalability is always restricted by the physical limits of computer systems while the option of horizontal scalability is both extensible and flexible as it facilitates the ability to easily scale out by adding storage space or adding a new physical machine. Hammoud et al. (2015) classified the data storage and query execution systems into four main paradigms, illustrated in Fig. 4, which are described as follows:

- **Paradigm-I**: Which represents the fully centralized option where the whole dataset (*D*) is absolutely stored on a single node and the evaluation of the user query (*Q*) is fully executed on the same node.
- **Paradigm-II**: Where the dataset (*D*) is distributed into *n* partitions $\{d_1, d_2, \ldots , d_n\}$ which are stored at *n* nodes while the evaluation of the user query (*Q*) is parallelized over the multiple partitions/nodes.
- **Paradigm-III**: Similar to Paradigm-II, the dataset (*D*) is distributed into *n* partitions $\{d_1, d_2, \ldots , d_n\}$ which are stored at different nodes, however, in this paradigm, the user query (*Q*) is decomposed into *m* sub-queries $\{q_1, q_2, \ldots , q_m\}$ where the evaluation of each sub-query $q_x$ is executed on one of the data partitions/nodes $d_y$.
- **Paradigm-IV**: In this paradigm, the dataset (*D*) is fully replicates at *n* nodes $\{D_1, D_2, \ldots , D_n\}$ while the user query (*Q*) is decomposed into *m* sub-queries $\{q_1, q_2, \ldots , q_m\}$ where the evaluation of each sub-query $q_x$ is executed on one of the data replicas/nodes $D_y$.

In principle, a main limitation on the *centralized* hybrid Disk/Memory representation, *Paradigm-I*, which has been proposed by Sakr et al. (2012) is its assumption that the graph topology may always fit to reside in the main memory of a single machine. Due to the continuous growth on the size of the graph datasets, this assumption might not be valid in many cases. In practice, currently, a single machine with a modern disk can still fit to host any big graph dataset (i.e., a graph with billions of nodes and edges), however, this may lead to severe thrashing to main memory and frequent accesses to disk. Consequently, this will lead to inefficient performance for any graph querying operations in addition to limited scalability. Therefore, managing big attributed graphs on a single machine may be infeasible, especially when the machine’s memory is dwarfed by the size of the graph topology (Hammoud et al. 2015). To overcome the limitations of centralized query engines, DG-SPARQL is designed as a distributed and scalable systems that takes the evaluation of G-SPARQL query to the next level. In particular, DG-SPARQL follows a variant strategy of *Paradigm-IV* where the attributed graph is encoded using the fully decomposition model, illustrated in Fig. 3, and fully replicated in a disk-based relational store across *n* nodes. In addition, the graph topology is partitioned across the main memory of the *n* nodes and encoded using a pointer-based representation. In DG-SPARQL, the user query (*Q*) is decomposed into *m* sub-queries $\{q_1, q_2, \ldots , q_m\}$ where the evaluation of each sub-query $q_x$ either can be pushed inside the relational store, via SQL, on one of the data replicas/nodes $D_y$ or evaluated via indexless memory-based graph traversal algorithms across the partitioned graph topology on the *n* nodes according rule-based and cost-based query optimization mechanism. As a result, DG-SPARQL can leverage larger aggregate memory capacities and higher computational power for managing attributed graph. More details about the distributed query execution mechanism will be presented in “Distributed Query Execution Engine” section.

In practice, by loading only the graph topology on the main memory of the underlying cluster, we are able to achieve a significant decrease on the main memory usage by avoiding the need to load the attributes of the graph node/edges and their data values, which are maintained in the $M + N$ attribute tables, into the main memory. This decrease in the memory usage enable greater scalability capabilities for managing bigger graphs on a defined memory size or reducing the number of the machines on the underlying computing cluster. In addition, this mechanism avoids building additional memory-based indices for the graph attributes that could be needed for improving the associated value-based query predicates and rely for such tasks on the well-designed optimization capacity of the underlying relational storage.

## Distributed query execution engine

### System architecture

**Fig. 5:**
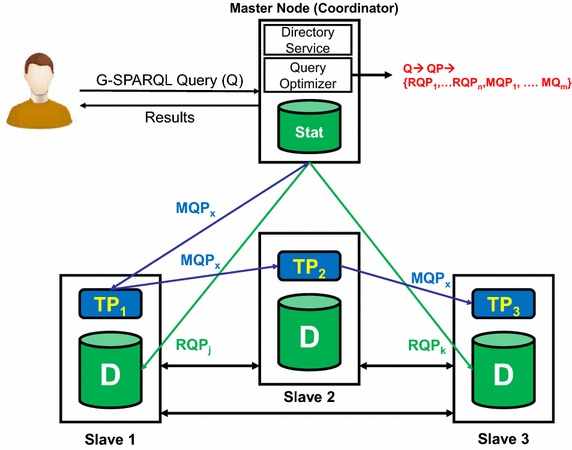
The architecture of DG-SPARQL query execution engine

Figure 5 illustrates the architecture of the DG-SPARQL query execution engine which follows the Master/Slave paradigm. In this architecture, one node is designated as the Master/Coordinator node which is responsible for query parsing, compilation, optimization and coordinating the query execution process. As described earlier, DG-SPARQL is designed to use multiple nodes/machines (slaves) to query big attributed graphs with topology structure information that can not be maintained in the main memory of a single node. In particular, let us assume that the DG-SPARQL underlying cluster contain *n* nodes. Then, the topology of the attributed graph is *partitioned* into *n* disjoint partitions where each partition (${ {TP}}_i$) is maintained in the main memory of one of the slave nodes while the relational encoding of the attributed graph is *replicated* on the relational store for all the *n* slave nodes. In this scheme, the coordinator node maintains the graph statistics information which is used during the query the optimization process. In addition, it uses a directory service that maintains two mappings: a mapping for each vertex identifier to its assigned memory partition identifier (${ {TP}}_i$) and a mapping for each edge identifier to its memory partition identifier (${ {TP}}_i$) as well. In general, the main goal of any effective graph partitioning algorithm is to preserve locality in graph accesses consequently to reduce communication overhead between partitions/nodes during the query evaluation process. In general, graph partitioning is a challenging problem by itself (Hendrickson and Kolda 2000) which is out of the focus of this work. In particular, DG-SPARQL remains agnostic towards the various graph partitioning schemes and is designed to be able to incorporate any of them. For our current implementation and experimental evaluation (“Experimental evaluation” section), we employed the METIS partitioner (METIS 2014). In practice, one of the advantages of the METIS partitioner is that it collocates the nearby vertices that are nearby to be collocated on the same partition/machine which reduces the communication cost of common graph traversal operations (e.g., BFS or DFS).

G-SPARQL is a declarative query language. Thus, for any G-SPARQL query, there are always various possible execution plans to evaluate such query. G-SPARQL is equipped with a query optimizer that seeks to optimize the query execution time for any input query. In particular, among a wide space of alternative possible query plans for executing the user input query, the query optimizer employs a cost model to predict the time execution cost of each plan then selects the execution plan that with the minimum cost for actual execution. In order to achieve this goal, the query coordinator node maintains a set of graph statistics (e.g., structural indices, selectivity information of value-based predicates on the attributes of graph nodes and edges) which are utilized by the query optimizer to estimate the time execution cost of each possible query plan. In practice, the query optimizer starts by compiling the user input query (*Q*) into a logical query plan $\textit{QP}$ using a defined set of G-SPARQL algebraic operators (Sakr et al. 2012). Using the statistical information and the cost model, the query optimizer compiles the logical query plan ($\textit{QP}$) into a set of sub-query physical query execution plans, $\textit{QP} \rightarrow \{\textit{RQP}_1, \ldots , \textit{RQP}_x, \textit{MQP}_1,\ldots , \textit{MQP}_y\}$, where each $\textit{RQP}_i$ refers to a relational-based sub-query plan which is to be evaluated by one of the relational store on the underlying *n* slave nodes via SQL queries, *x* refers to the number of relational-based sub-query plans which is less than or equal to the number of slave machines (*n*) and each $\textit{MQP}_j$ refers to a main memory query plan which is to be evaluated via graph traversal operations.

DG-SPARQL is designed to evaluate the sub-query plans in a parallel fashion. In particular, DG-SPARQL parallelizes the evaluation of the *x* relational-based sub-query plans by assigning the evaluation of each plan $\textit{RQP}_i$ into a distinct relational store of the underlying slave nodes (*n*). In addition, DG-SPARQL parallelizes the evaluation of the main memory query plans by relying on Bulk Synchronous Parallel-based (Valiant 1990) graph traversal operations and communication over the graph partitions $(TP_n)$. In the following subsection, we present the query optimization and execution details of the DG-SPARQL query engine.

### Query optimization and execution in DG-SPARQL

In general, one of the powerful features of any declarative query language, like G-SPARQL, is that it provides its users with the ability to describe the logic of their querying operation without the need to get into the details of how such query will be executed. In particular, it becomes the responsibility of the query execution engine to enumerate the various possible query execution plans, for any user declarative query, and select among them one for actual execution. Ideally, the selected plan is the one with the lowest execution time. In practice, choosing such an optimal execution plan is not a trivial task. Therefore, DG-SPARQL relies on a set of cost-based query optimization techniques that attempt to estimate the cost of the various possible execution plans and predicts the one with the lowest-cost or at least a closest one to it. In order to achieve this goal, DG-SPARQL starts by compiling the user input query into a logical query plan using a defined set of G-SPARQL algebraic operators, listed in Table 1 (Sakr et al. 2012). In general, the G-SPARQL algebraic operators can be classified into two main groups:

- *Retrieval-Based Operators*: This group of operators (NgetAttVal, EgetAttVal, getEdgeNodes, strucPred, edgeJoin) is mainly used for retrieving a target set of the graph nodes and edges and can be intuitively represented by the standard relational operators (e.g., select, project, join) (Sakr et al. 2012).
- *Traversal-Based Operators*: This group of operators (pathJoin, sPathJoin, filterPath) is mainly evaluated via traversal operations over the graph topology and can not be intuitively represented by the standard relational operators.

**Table 1:** *G-SPARQL* algebraic operators Sakr et al. (2012)

Operator	Description
NgetAttVal	Returns the values of an attribute for a set of nodes
EgetAttVal	Returns the values of an attribute for a set of edges
getEdgeNodes	Returns adjacent nodes, optionally through a specific relation, for a set of graph nodes
strucPred	Returns a set of vertices that are adjacent to other vertices with a specific relationship and optionally returns the connecting edges
edgeJoin	Returns pairs of vertices that are connected with an edge, optionally of a specified relationship, and optionally returns the connecting edges
pathJoin	Returns pairs of vertices which are connected by a sequence of edges of any length, optionally with a specified relationship, and optionally returns connecting paths
sPathJoin	Returns pairs of vertices which are connected by a sequence of edges of any length, optionally with a specified relationship, and returns the *shortest* connecting path
filterPath	Returns paths that satisfy a condition

**Fig. 6:**
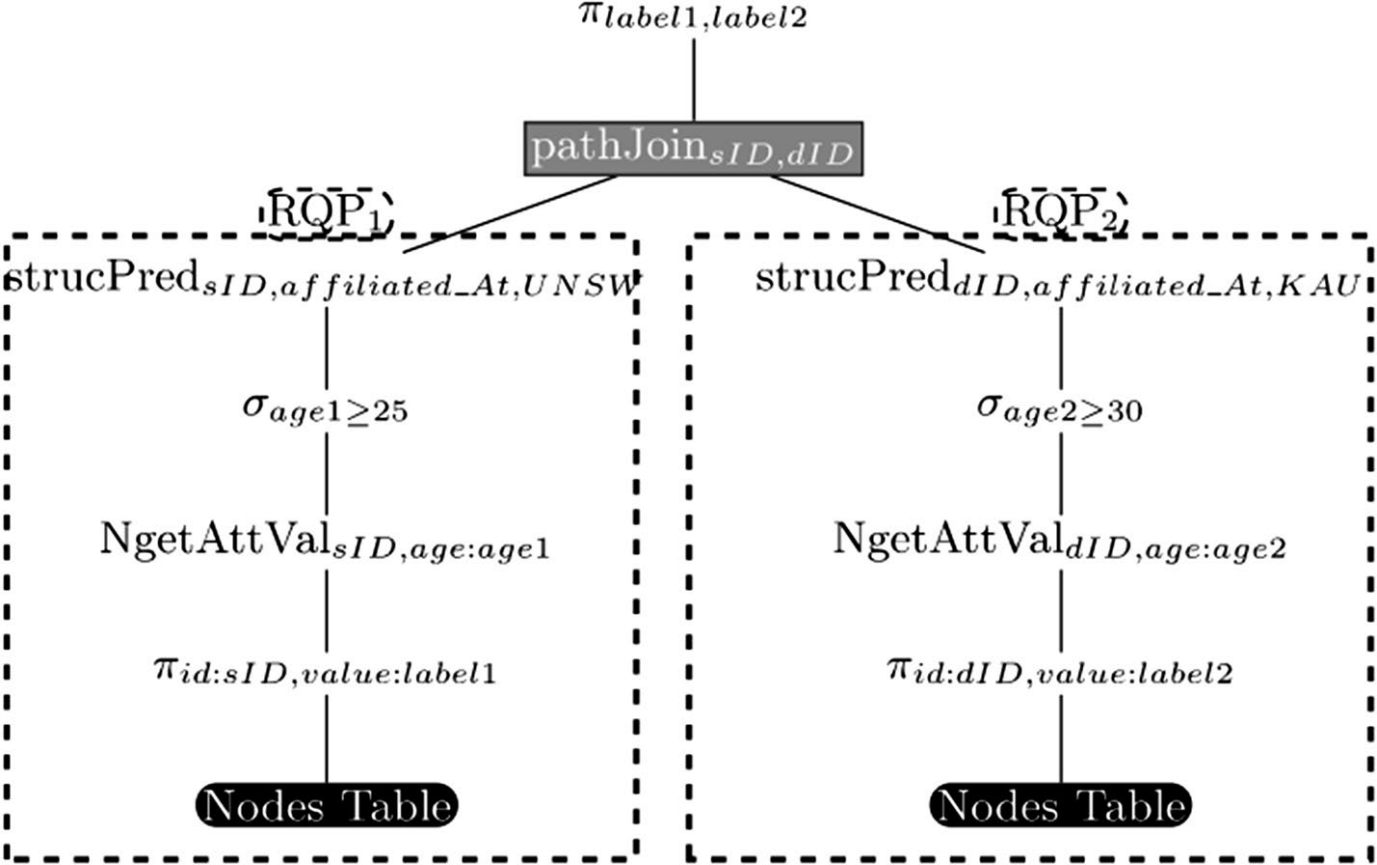
An example DAG plan for G-SPARQL

After generating the initial logical plan of the input G-SPARQL query, this initial plan gets optimized using some common rules that include the traditional rules for relational algebraic optimization (e.g. pushing the selection operators down the plan) in addition to some rules that are specific to the context of the G-SPARQL query plans (Sakr et al. 2014). To illustrate, Fig. 6 presents an example algebraic compilation for the following G-SPARQL query:

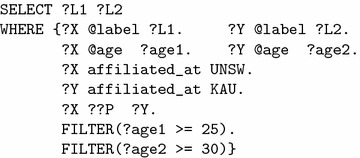



As illustrated in Fig. 6, G-SPARQL logical plans are commonly organized in a DAG shape. In particular, the query planner traverses the logical query plan in a bottom-up fashion (starting by the leave nodes and then climbing the various paths of the query plan up to the root) and groups the connected neighbours retrieval-based operators into initial set of relational-based sub query plans (*RQP*). This climbing process for each path stops once it touches a traversal-based operator. For example, in Fig. 6, as indicated by dashed rectangles in the figure, two candidate relational-based sub-query plans, $\textit{RQP}_1$ and $\textit{RQP}_2$, have been identified. In principle, the main strategy of DG-SPARQL query execution engine is to group the retrieval-based operators into relational-based sub-query plans (*RQP*) and parallelize their execution, via SQL queries, using the relational stores of the underlying nodes while each traversal-based operators represents a main memory query plan (*MQP*) which is evaluated using BSP-based traversals (Valiant 1990), synchronized by the coordinator node, over the partitioned graph topology. Using this mechanism, DG-SPARQL can rely on the underlying relational stores on finding the most efficient physical execution plan of its assigned sub-query plans, represented via SQL queries, by leveraging its built-in advanced and sophisticated query optimization mechanisms (e.g., join ordering, join implementation, index selection).

To illustrate the performance advantage of DG-SPARQL as a distributed and parallel query execution engine, let us consider the illustrated query plan of Fig. 6. For such query plan, the query evaluation process using a centralized execution engine of G-SPARQL typically goes through a sequential execution of the following three main steps:

1. S1: The relational-based sub-query plan ($\textit{RQP}_1$) gets translated into SQL query ($\textit{SQL}_1$) which is pushed for evaluation inside the underlying relational store.
2. S2: The relational-based sub-query plan ($\textit{RQP}_2$) gets translated into SQL query ($\textit{SQL}_2$) which is pushed for evaluation inside the underlying relational store.
3. S2: The results, retrieved nodes, of ($\textit{SQL}_1$) and ($\textit{SQL}_2$) are then passed for further memory-based processing using the traversal-based operator, pathJoin, and the following operators in the query plan.

As a result, the total execution time for this execution plan in a centralized query engine (*CentralizedT*) is represented by the sum of the execution times of the three steps:

$$\begin{aligned} \textit{CentralizedT} = T(S1) + T(S2) + T(S3) \end{aligned}$$

On the other hand, a simple intuitive alternative execution plan in the DG-SPARQL execution engine is to parallelize the execution of ($\textit{SQL}_1$) and ($\textit{SQL}_2$), representing the two steps S1 and S2, over the relational stores of two distinct slave machines, to retrieve the target graph vertices and edges, and then parallelize the execution of the pathJoin operator over the partitioned graph topology, (S3). Therefore, the total execution time for this parallelized execution plan in DG-SPARQL (*DistributedT*) can be represented as follows:

$$\begin{aligned} \textit{DistributedT} = Max\,(T(S1), T(S2)) + T(P(S3)) \end{aligned}$$

where (*P*(*S*3)) represents the BSP-based parallel implementation of (S3). Clearly, the parallel execution of G-SPARQL query plans using DG-SPARQL mechanism can show a significant reduction in the total execution time.

In practice, in DG-SPARQL, the execution of each candidate relational-based sub query plan (*RQP*) can typically have various alternatives. For example, it can be translated into a single SQL query which is executed by a single relational store of the underlying nodes. Alternatively, it can be decomposed and translated into multiple SQL queries which are parallelly executed over multiple nodes. It is the job of the G-SPARQL query optimizer to enumerate the various possible decompositions ($D_1,D_2,\ldots ,D_v$) for the candidate relational-based query plan and chooses only one plan, which is predicated to have the lowest execution time, for actual execution. For example, let us assume a relational-based sub query plan ($\textit{RQP}$) with a possible decomposition ($D_i$) into the set of decomposed plans, $\textit{RQP}\rightarrow \{\textit{DRQP}_1, \textit{DRQP}_2, \ldots , \textit{DRQP}_d\}$. The DG-SPARQL predicts the total execution time ($TT(D_i)$) of each possible decomposition by estimating the following components:

- $ET(\textit{DRQP}_x)$: represents the estimated execution time for locally evaluating any decomposed plan, $\textit{DRQP}_x$, on its assigned slave node to return its intermediate result of size $\textit{IRRQP}_x$.
- $\textit{MT}(\textit{DRQP}_{xy})$: represents the required time to move (transfer) intermediate results from a node executing a decomposed plan $\textit{DRQP}_x$ into a node executing the decomposed plan $\textit{DRQP}_y$. In practice, the cost of data transfer is a dominant factor in any distributed system, therefore, the DG-SPARQL query optimizer typically chooses to move the data from the node with the smaller intermediate result size to the node with the highest intermediate result in order to reduce the data transfer cost. In other words, $\textit{MT}(\textit{DRQP}_{xy}) = min (\textit{IRRQP}_x, \textit{IRRQP}_y)$.
- $\textit{JT}(\textit{RQP}_{xy})$: represents the time to join two intermediate results produced by the two decomposed plans $\textit{DRQP}_x$ and $\textit{DRQP}_y$.

In practice, all decomposed plans would initially run in parallel, however, depending on the dependency graph between the decomposed plans, some of the decomposed plan are not able to start joining their intermediate results with the intermediate result of an external node until it finishes the generation of its intermediate results and is receiving the intermediate results from the external node. As a result, using the dependency graphs of the decomposition plans and the estimated costs for its various components, the query optimizer can estimate the total execution times of each decomposition plan, $\textit{TT}(D_i)$, and choose the plan with the lowest cost for actual execution. It should be also noted that the query optimizer takes into consideration the number of available nodes for the various relational-based query plans and their various associated decompositions in a way that the total number of decomposed plans of all relational-based query plans should not exceed the number of the available (*n*) relational stores of the slave nodes.

In principle, the basic implementation of the DG-SPARQL query execution engine relies on BSP-based main memory traversal algorithms for evaluating the traversal-based operators (e.g., reachability and shortest path operators). However, it should be noted that the DG-SPARQL query execution engine remains agnostic to the physical execution of the logical traversal operators and is able leverage any available indexing information to improve the query evaluation process of the different types of queries by taking into consideration the trade-off of building and maintaining their indices in addition to their main memory consumption. For example, distributed graph indexing and query answering techniques (Fan et al. 2012) can be leveraged for accelerating the execution of the traversal-based operators. However, such indexing methods can be only considered in the cases where there are no restrictions or conditions on the nodes and edges of the results of the operators as these indexing methods usually do not consider such filtering or predicate evaluation functionalities in their design.

## Experimental evaluation

We implemented DG-SPARQL using C++ and MPICH,^3^ a high performance implementation of the Message Passing Interface (MPI). The implementation includes the query language parser and compiler, cost-based query optimizer, and distributed query execution engine. We used the IBM DB2 RDBMS for storage, indexing and performing all SQL-based query evaluation. We implemented a BSP-based version of the breadth-first graph traversal algorithm which is used for evaluating our traversal-based reachability and shortest path operators (Redekopp et al. 2013). In this section, we present our experimental evaluation for DG-SPARQL. The main objective of our experimental evaluation is to assess two main aspects: the system performance scalability on handling big attributed graphs in addition to comparing the system performance with Apache Giraph,^4^ a popular distributed graph processing system which is built on top of the Hadoop framework.^5^

### Experimental setup

#### Experimental environment

Our experiments have been conducted on a cluster of 20 nodes in addition to one node that servers as the system coordinator and client. Each server has an Intel QuadCore 2.9 GHz CPU, 16 GB of main memory storage, 1 TB of SCSI secondary storage and runs the 64-bit Fedora 13 Linux operating system, MPICH 3.0.4. For the comparison with Apache Giraph Systems, we have been using Apache Hadoop 2.6.0, Apache Giraph 1.1.0 and Java version 7.

#### Dataset

In our experiments, we used two main datasets:

- The popular LUBM benchmark (2014) which provides an ontology for academic information (e.g., universities). This is a *synthetic* dataset that can be generated with various sizes by controlling the number of generated universities. The original data generator of the benchmark generates the dataset according to the RDF graph model. Therefore, we have modified the data generator of the benchmark to generate the dataset according to the attributed graph model.^6^ In order to evaluate the scalability of our system, we generated four datasets at different scales with 20K (D1), 30K (D2), 40K (D3), and 50K (D4) universities with 450 GB, 700 GB, 950 GB and 1.2 TB of data, respectively. The datasets have been partitioned across the 20 nodes using the METIS partitioner (METIS 2014).
- The *real* DBpedia 3.8 dataset.^7^ We converted the RDF data model of this dataset into a property graph data model using the following mechanism (Sun et al. 2015):
  - Each subject or object node in the RDF graph becomes a vertex with a unique integer ID in the property graph.
  - Object properties in the RDF graph are represented as adjacency edges in the property graph, where the source and the target of the edge were vertex IDs, and the edge was identified by an integer ID.
  - Datatype properties in the RDF graph were are represented as attributes in the property graph.
  - Provenance or context information are represented as attributes for the graph edges.

For the sake of measuring the performance speed-up of the query execution time in response to increasing the number of slave machines, we have partitioned this dataset three times into 2, 4 and 6 partitions using the METIS partitioner (METIS 2014).

#### Workload

In practice, there is no defined standard benchmarks for evaluating the performance of query engines for attributed graphs (Sakr et al. 2014). Therefore, we defined four main categories of attributed graph queries which we used in our evaluation. These categories are described as follows:

- QT1—*Highly Selective Pattern Matching Queries*: This category represents a connected graph pattern (e.g., path, star, subgraph) with highly selective predicates that matches to a small set of answers.
- QT2—*Low Selective Pattern Matching Queries*: This category represents a connected graph pattern with low selective predicates that matches to a large set of answers.
- QT3—*Pattern Matching Queries Combined With Traversal-Based Operators*: Combines graph pattern searches with one or more traversal-based operators (e.g., rechability checks, shortest path).
- QT4—*Pattern Matching Queries Combined With Traversal-Based Operators and Path Filtering Operations*: Combines graph pattern searches with one or more traversal-based operators in addition to applying filtering predicates on the traversed paths by the traversal-based operators.

For each query type, we assigned random literal values of the query templates in order to generate different query instances. Each query template is instantiated 20 times where the data values are generated randomly.

#### Performance evaluation metric

Our main performance metric is the query execution time.

In particular, each query instantiation of the experimental workload has been executed 5 times over our implementation and the Apache Giraph system, and execution times were collected. All times are in seconds. In order to to ensure that any caching or system process activity would not affect the collected results, the longest and shortest times for each instantiation were dropped and the remaining three execution times for the 20 instantiations were averaged.

### Experimental results

**Fig. 7:**
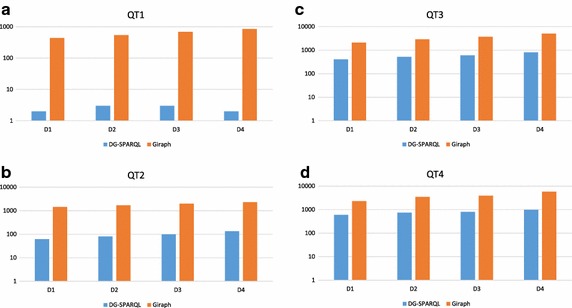
Average query execution times of DG-SPARQL VS Giraph on LUMB datasets **a** Query Type QT1 **b** Query Type QT2 **c** Query Type QT3 **d** Query Type QT4

Figure 7 illustrates the average query execution times on DG-SPARQL and Giraph for the 20 instances of each of the identified four query types on the four experimental datasets of the LUMB benchmark. The results of the experiments show that DG-SPARQL is able to outperform Giraph with orders of magnitudes on the various query types. It also shows that DG-SPARQL is able to scale well to handle the increasing datasets of the LUMB benchmark.

To better understand the underlying factors for the performance improvement of DG-SPARQL over Giraph, we looked closer at the details of each query. In particular, for QT1 (Fig. 7a), highly selective pattern matching queries, the main strategy of DG-SPARQL is to translate the query plan of this type of queries into an SQL query which is *centrally* executed on one of the underlying RDBMS nodes. In principle, this type of query does not involve any graph traversal operations, therefore, it does not require any message exchange between the cluster nodes. In addition, due to the high selectivity of its query predicates, RDBMS can effectively utilize its solid indexing infrastructure for efficient evaluation. Therefore, for this type of query, DG-SPARQL has shown the highest order of magnitudes in performance improvement of G-SPARQL over Giraph. Additionally, the percentage of performance improvement has increased as the size of the processed graph dataset increased.

For QT2 (Fig. 7b), low selective pattern matching queries, the strategy of DG-SPARQL is to translate the query plan of this type of queries into an SQL query with a *distributed* execution plan on more than one of the underlying RDBMS nodes. Similar to QT1, QT2 does not involve any graph traversal operations, however, due to the distributed execution of the SQL queries, the evaluation of such a query type requires some form of message exchange and data shuffle operation to be performed between the nodes of the cluster which are involved in the query evaluation. Thus, for this type of queries, DG-SPARQL is still able to scale better and outperforms Giraph with orders of magnitudes, however, the percentage of improvement on QT2 is lower than the percentage of improvement on QT1, which is centrally executed and does not involve any network communication overhead.

QT3 (Fig. 7c) combines pattern matching operations with traversal-based operations, thus, DG-SPARQL splits the query execution plan for this type of queries into multiple sub-plans where some of these plans are represented as SQL queries and their execution is pushed to the underlying RDBMS nodes while some other plans are evaluated using the BSP-based BFS traversal (Redekopp et al. 2013). Based on the cost model, the execution of each SQL-based query plan can be centralized on a single RDBMS node or distributed over more than one node. The same strategy is applied for the queries which belong to query type, QT4 (Fig. 7d). The main difference between query types, QT3 and QT4, is the execution of the path filtering condition on QT4 requires a post-processing step, after determining all the connecting paths between each pair of vertices, to evaluate the filtering predicates on the retrieved paths. For both query types, DG-SPARQL is also able to scale better and outperforms Giraph with order of magnitudes, however, the percentage of improvement on QT4 is the lowest among the four query types due to the network communication overhead and the post processing steps.

**Fig. 8:**
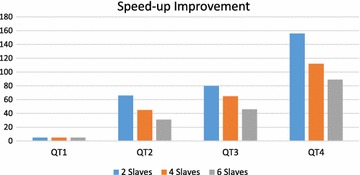
Speed-up improvement of query execution time in response to increasing the number of slave nodes (partitions)

Figure 8 illustrates the average query execution times on DG-SPARQL for 20 instances of each of the identified four query types on the DBpedia real dataset. In order to evaluate the speed-up improvement of query execution time in response to increasing the number of slave nodes (partitions), we have repeated this experiment three times using 2, 4 and 6 slave nodes. The results of the experiments show that the percentage of speedup improvement varies from one query type to another. For example, for QT1, the query plan of this type of queries is translated into a single SQL query which is centrally executed on one of the underlying RDBMS nodes. Therefore, increasing the number of underlying slave nodes does not introduce any speedup improvement for the query execution time of such type of queries. Queries of query type QT2 achieve the highest percentage of speedup improvement by increasing the number of the underlying slave nodes due to their *distributed* execution plans over the underlying RDBMS nodes. In particular, increasing the number of slave nodes from 2 to 4 leads to 34 % speedup improvement while increasing the number of slave nodes from 2 to 6 leads to 59 % speedup improvement on the query execution times. Queries of query types QT3 and QT4 achieve comparable percentage of speedup improvement by increasing the number of the underlying slave nodes due to due to the network communication overhead and the post processing steps of their query plans. In particular, for QT3, increasing the number of slave nodes from 2 to 4 leads to 24 % speedup improvement while increasing the number of slave nodes from 2 to 6 leads to 43 % speedup improvement on the query execution times. For QT4, increasing the number of slave nodes from 2 to 4 leads to 25 % speedup improvement while increasing the number of slave nodes from 2 to 6 leads to 44 % speedup improvement on the query execution times. It should be noted that increasing the number of slave nodes does not necessarily lead to increasing the speedup improvement on the query execution times as the number of relational-based sub-query plans can be less than the number of slave machines. In this case, increasing the number of slave nodes does not lead to speedup improvement on the query execution time for this type of queries. In addition, increasing the number of slave nodes leads to an increase in the overhead of the message exchange between the cluster nodes. This overhead affects having a linear relationship between the number of slave nodes and query execution time.

## Related work

Several languages were introduced for querying various kinds of graph models with various aims and querying constructs. For instance, *GraphQL* (He and Singh 2008) is a graph query language that relies on graph patterns as the fundamental querying units. The language design has mainly focused on manipulating and querying labeled directed graphs. The *GraphDB* (Güting 1994) language has been designed to support spatial networks (e.g., transportation systems) based on the availability of a graph schema. GraphDB querying abstractions rely on object-oriented concepts including classes for vertices, edges and paths. It supports regular expressions that are specified over sequences of vertex and edge types. SoQL (social networks query language) (Ronen and Shmueli 2009) has been presented as an SQL-like language for querying social networks. SoQL provides its users with the ability to retrieve paths and use these retrieved paths to create new connections with the retrieved nodes which are located at the end of the paths. The language also enables to formulate complex conditions over the retrieved paths. *PQL* (Leser 2005) has been designed as a special-purpose language that is focused around querying pathways of biological networks.

*GRAPHiQL* (Jindal and Madden 2014) has been introduced as another SQL-like general purpose graph processing language. GRAPHiQL provides its user with the ability to reason about graphs in terms of the intuitive abstraction of vertices and edges. It also provides optimized graph querying constructs such as recursion, looping, neighborhood access. The GRAPHiQL execution engine compiles the user query into SQL query that is executed by a standard relational engine and relies on query optimization techniques to tune the performance of these queries. *Green-Marl* (Hong et al. 2012) has been presented as a domain-specific language (DSL) with high level language constructs that enables its users to express their graph querying operations. The execution engine of Green-Marl translates the user programs into efficient C++ code that exploits data level parallelism and the high-level semantic knowledge of the language constructs. *G-Path* (Bai et al. 2013) has been introduced as a path-based query language on large graphs. The execution engine of G-Path is designed on top of the Hadoop framework (Sakr et al. 2013) and bulk synchronized parallel model (Batarfi et al. 2015) where it executes general graph queries in the absence of any indexing information. *Gremlin* (2015), *Cypher* (2015) and *Horton* (Sarwat et al. 2012) are examples of other path-based languages which are used for graph traversals. *Horton+* (Sarwat et al. 2013) has been implemented as a distributed execution engine for Horton queries that fully manages the graph using the main memory of a cluster of nodes. In practice, path-based languages may limit the ability of its users to only think in terms of paths and constrain their ability to express broader graph querying operations. *SQLGraph* (Sun et al. 2015) has been presented as an approach that exploits both relational and non-relational storage for property graphs. In particular, it uses relational storage for adjacency information and JSON storage for vertex and edge attributes. SQLGraph applies a query translation mechanism that translates Gremlin queries (2015) into SQL queries and leverage relational query optimizers and execution engines for evaluating the queries. *GRAPHITE* (Chau et al. 2008) has been presented as a visual system for querying graph patterns and locates both exact and approximate subgraph matches in large attributed graphs. *VOGUE* (Bhowmick et al. 2013) is another visual human computer interaction(HCI)-aware subgraph query engines that interleaves visual query construction and query processing with the aim of improving the user experience and performance of query execution.

For about a decade, the Hadoop framework has often been considered as the de facto standard in the domain of general distributed computation and big data processing (Sakr et al. 2013). In general, the MapReduce programming model of the Hadoop framework is able to implement many common graph querying and processing operations. However, the Hadoop framework has shown to have limited practicality in the context of big graph processing. In practice, graph processing algorithms are mostly iterative in nature and require the traversal of the graph in a particular form. Using the Hadoop framework, this could be implemented via a sequence of job invocations which passes the whole state of the graph from one step to the following. However, such mechanism is not adequate for graph processing and leads to inefficient performance because of the associated serialization and communication overheads (Batarfi et al. 2015). To solve this inherent performance problem of the Hadoop framework, several specialized platforms which are designed to serve the unique processing requirements of large-scale graph processing have been introduced. These systems provide programmatic abstractions for performing iterative parallel analysis of large graphs on clustered systems (Batarfi et al. 2015).

In general, vertex-centric models express the graph processing job from a vertex perspective where they are executed iteratively for each vertex in the graph. The *Pregel* system (Malewicz et al. 2010), introduced by Google, has pioneered the domain of large scale graph processing systems using the Bulk Synchronous Parallel (BSP) programming model and by relying on a “*think like a vertex*” programming model. The introduction of Google’s Pregel has triggered a lot of interest in the domain of large-scale graph processing and inspired the development of several Pregel-based systems which have been attempting to exploit different optimization opportunities. For example, *Apache Giraph*^8^ is an open source project that clones the ideas and implementation of the Pregel specification in Java on top of the infrastructure of the Hadoop framework. *GPS* (Salihoglu and Widom 2013), and *Giraph*++ (Tian et al. 2013) are examples of other systems that have been presented as enhancements/extensions for the Pregel system in various aspects. *Trinity* (Shao et al. 2013) is a memory-based distributed system with the aims of optimizing memory and communication cost under the assumption that the entire graph is partitioned over a memory cloud. *GraphX* (Gonzalez et al. 2014) is a distributed graph processing system which is implemented on top of the Spark framework (Zaharia et al. 2010). Other general purpose distributed graph processing systems include *Pregelix* (Bu et al. 2014), *GRACE* (Wang et al. 2013), *NScale* (Quamar et al. 2014), *GraphLab* (Low et al. 2012) and *PowerGraph* (Gonzalez et al. 2012). In general, these group of systems are mainly designed for batch processing of large scale graph computations rather than online graph querying. In addition, they lack any declarative interfaces and thus they require their users to be experienced programmers to write efficient programs that acknowledge deep understanding of the programming model and the underlying system details.

In addition to the distributed graph processing platforms, *NXgraph* (Chi et al. 2015), *GraphChi* (Kyrola et al. 2012) and *TurboGraph* (Han et al. 2013) have been presented as *centralized* systems to process large graphs that are stored in the secondary storage of a single node. However, several experimental studies have shown the performance and scalability limitations of the centralized graph processing systems (Barnawi et al. 2014), (Koch et al. 2015). Several centralized (Abadi et al. 2007; Bröcheler et al. 2009; Neumann and Weikum 2008; Zou et al. 2011) and distributed (Hammoud et al. 2015; Rohloff and Schantz 2010; Jiewen et al. 2011; Zeng et al. 2013; Papailiou et al. 2013) SPARQL query engines for the RDF graph data model have been proposed. However, these systems can not be directly reused in the context of attributed graph because of the various differences in the data model and querying requirements. Moreover, several centralized graph database systems (e.g. Neo4j,^9^ HypergraphDB^10^) have also been introduced. However, such systems can not scale to deal with the performance requirements of querying large graphs.

## Conclusion

In this article, we presented DG-SPARQL, an efficient distributed query engine for the G-SPARQL query language which is able to handle big attributed graphs. DG-SPARQL relies on an efficient hybrid Memory/Disk representation of large attributed graphs where only the topology of the graph is maintained in the distributed memory of computing clusters while the data of the graph are stored in a relational database. The DG-SPARQL query execution engine relies on a cost model to split the execution plan into relational-based plans and main memory-based plans. In addition, using the cost model, the query execution engine can adaptively switch the execution of the plans between being centralized or distributed based on which is the more efficient model. Our experimental evaluation validated the efficiency and scalability of our approach and showed that DG-SPARQL is a scalable engine that works for massive graphs. Due to the complexity of graph query languages, in our future work, we are planning to support visual query interfaces (Hung et al. 2014) that can reduce the burden of query formulation and ease the process for different types of non-technical users.

## Footnotes

1 http://venturebeat.com/2015/04/22/facebook-passes-1-44b-monthly-active-users-1-25b-mobile-users-and-936-million-daily-users/.

2 Throughout the paper, we use the words node and vertex interchangeably.

3 https://www.mpich.org/.

4 http://giraph.apache.org/.

5 https://hadoop.apache.org/.

6 The main extension is to generate attribute/value pairs for the graph edges.

7 http://wiki.dbpedia.org/services-resources/datasets/data-set-38/downloads-38.

8 http://giraph.apache.org/.

9 http://neo4j.org/.

10 http://www.kobrix.com/hgdb.jsp.
